# Aerobic Interval Training Impacts Muscle and Brain Oxygenation Responses to Incremental Exercise

**DOI:** 10.3389/fphys.2019.01195

**Published:** 2019-09-20

**Authors:** Kevin Caen, Kobe Vermeire, Silvia Pogliaghi, Annelies Moerman, Victor Niemeijer, Jan Gustaaf Bourgois, Jan Boone

**Affiliations:** ^1^Department of Movement and Sports Sciences, Ghent University, Ghent, Belgium; ^2^Department of Neurosciences, Biomedicine and Movement Sciences, University of Verona, Verona, Italy; ^3^Department of Anesthesiology, Ghent University Hospital, Ghent, Belgium; ^4^Department of Sports Medicine, Elkerliek Hospital, Helmond, Netherlands

**Keywords:** incremental ramp exercise, interval training, cerebral oxygenation, muscle oxygenation, limiting factors

## Abstract

The purpose of the present study was to assess the effects of aerobic interval training on muscle and brain oxygenation to incremental ramp exercise. Eleven physically active subjects performed a 6-week interval training period, proceeded and followed by an incremental ramp exercise to exhaustion (25 W min^–1^). Throughout the tests pulmonary gas exchange and muscle (Vastus Lateralis) and brain (prefrontal cortex) oxygenation [concentration of deoxygenated and oxygenated hemoglobin, HHb and O_2_Hb, and tissue oxygenation index (TOI)] were continuously recorded. Following the training intervention $\dot{\text{V}}\,{\text{O}}_{2}$_peak_ had increased with 7.8 ± 5.0% (*P* < 0.001). The slope of the decrease in muscle TOI had decreased (*P* = 0.017) 16.6 ± 6.4% and the amplitude of muscle HHb and totHb had increased (*P* < 0.001) 40.4 ± 15.8 and 125.3 ± 43.1%, respectively. The amplitude of brain O_2_Hb and totHb had increased (*P* < 0.05) 40.1 ± 18.7 and 26.8 ± 13.6%, respectively. The training intervention shifted breakpoints in muscle HHb, totHb and TOI, and brain O_2_Hb, HHb, totHb and TOI to a higher absolute work rate and $\dot{\text{V}}\,{\text{O}}_{2}$ (*P* < 0.05). The relative (in %) change in $\dot{\text{V}}\,{\text{O}}_{2}$_peak_ was significantly correlated to relative (in %) change slope of muscle TOI (*r* = 0.69, *P* = 0.011) and amplitude of muscle HHb (*r* = 0.72, *P* = 0.003) and totHb (*r* = 0.52, *P* = 0.021), but not to changes in brain oxygenation. These results indicate that interval training affects both muscle and brain oxygenation, coinciding with an increase in aerobic fitness (i.e., $\dot{\text{V}}\,{\text{O}}_{2}$_peak_). The relation between the change in $\dot{\text{V}}\,{\text{O}}_{2}$_peak_ and muscle but not brain oxygenation suggests that brain oxygenation *per se* is not a primary factor limiting exercise tolerance during incremental exercise.

## Introduction

Incremental ramp exercise tests are commonly used in healthy and pathologic populations to determine physical fitness, to identify intensity zones for training/rehabilitation, and to assess the efficacy of training and/or treatment interventions. Next to the measurement of pulmonary gas exchange, which provides insight into whole-body physiological responses to increasing exercise intensity, also peripheral measures of regional oxygenation (predominantly at the level of the locomotor muscles) have been performed in the recent past. More specifically, the signals [the concentration of oxygenated (O_2_Hb) and deoxygenated hemoglobin (HHb)] derived from near-infrared spectroscopy reflect the relationship between O_2_ delivery and O_2_ utilization at the level of the microcirculation (Mancini et al., 1994). As such, more specific information can be obtained on the peripheral physiological responses and on the limiting factors of different populations to incremental exercise.

The NIRS responses at the level of the locomotor muscles have already been characterized extensively (for review see Boone et al., 2016) in healthy subjects, trained subjects, children, elderly and some patient populations. Traditionally, it is observed that muscle HHb, which is often considered as a reflection of fractional O_2_ extraction (Delorey et al., 2003; Grassi et al., 2003), increases following a sigmoid-like pattern, with a sluggish increase at the onset of the incremental exercise, followed by a linear increase to finally reach a plateau at high intensities (∼80–90%VO_2__max_) (Ferreira et al., 2007; Spencer et al., 2012; Fontana et al., 2015). In this context, it has been shown from cross-sectional studies that trained subjects have a less steep increase but higher peak value in HHb, reflecting an improved matching between O_2_ supply and O_2_ demand and higher peak O_2_ extraction, respectively (Boone et al., 2009; Gifford et al., 2016; Okushima et al., 2016). The longitudinal effects of a training intervention on muscle oxygenation are scarcely documented. Prieur and Mucci (2013) found an increased amplitude of the HHb response (i.e., indicating an improved O_2_ extraction capacity) following 6 weeks of interval training at high intensities. Takagi et al. (2016) found a similar impact on the amplitude of HHb following an aerobic training program in post-myocardial infarction patients.

Recently, also oxygenation responses to incremental exercise at the level of the brain have gained interest since it has been argued that the brain might be involved in the process of termination of maximal exercise (Robertson and Marino, 2016), especially since Nielsen et al. (Nielsen et al., 1999) found that O_2_ supplementation could maintain cerebral oxygenation at a higher level and as such increased performance. In healthy subjects cerebral oxygenation (cO_2_Hb) increases steadily during incremental exercise compared to baseline resting levels (Rooks et al., 2010). However, at high intensities (in close proximity to the respiratory compensation point) cO_2_Hb levels-off and even decreases (Bhambhani et al., 2007; Racinais et al., 2014; Oussaidene et al., 2015). In this context, it has even been shown that neural activity in the prefrontal cortex decreases at the respiratory compensation point (Robertson and Marino, 2015). Cross-sectional data show that trained subjects have a more pronounced increase in cO_2_Hb, indicating an improved cerebral oxygenation compared to less trained counterparts, which might add to the training-induced improvement in exercise tolerance (Rooks et al., 2010; Oussaidene et al., 2015). However, it is currently unclear whether cerebral oxygenation responses, as assessed in cO_2_Hb measured with NIRS, to incremental exercise are affected by short-term training interventions.

Therefore, the purpose of the present study was to assess the effects of a 6-week interval training program on muscle and cerebral oxygenation responses to incremental exercise. In line with cross-sectional studies we hypothesize first, that the amplitude of mHHb will have increased (as a reflection of an improved O_2_ extraction capacity) and that the slope of the increase in mHHb relative to work rate will be lower (as a reflection of improved matching between O_2_ supply and O_2_ demand) following the training intervention. Second, we hypothesized that the amplitude of cO_2_Hb will have increased (as a reflection of an improved cerebral oxygenation) and the leveling-off will occur at a higher absolute work rate, since it is has been proposed that the breakpoint in cO_2_Hb is mechanistically linked to the respiratory compensation point (Bhambhani et al., 2007; Racinais et al., 2014). Third, given the suggestion that cerebral oxygenation possibly affects exercise tolerance and the observation that cerebral oxygenation is improved in trained subjects, we will assess whether changes in muscle and/or cerebral oxygenation following the training intervention contribute to the improvement in VO_2__peak_ to obtain insight into the limiting factors of incremental ramp exercise.

## Materials and Methods

### Ethics Statement

This study was approved by the local ethical committee (Ghent University Hospital, Ghent, Belgium) with the code number EC/2015/1318 and followed the ethical recommendations for the study of humans as suggested by the Declaration of Helsinki. All participants give written informed consent prior to the start of the study.

### Subjects

Eleven male physically active students (21.8 ± 1.2 year, 1.81 ± 0.08 m, 75.7 ± 4.0 kg) volunteered to take part in this study. Based on the effect (Cohen’s *d*: 0.8–2.0) of training interventions on the available NIRS responses (Prieur and Mucci, 2013; Takagi et al., 2016) a subject group of 5–11 subjects would be sufficient to obtain a power of 0.80 with an α of 0.05. All subjects participated in various recreational sports activities on a regular basis (1–3 times per week) and were habituated to maximal exercise efforts, although none of them had a history of cycling training. Prior to the study each participant underwent a medical examination. Each subject was declared to be in good health and no contra-indications for participation were detected.

### Experimental Procedure

#### General Overview

Experimental testing (i.e., incremental ramp exercise) was conducted on an electromagnetically braked cycle ergometer (Lode Excalibur Sport, Groningen, Netherlands) and took place in the laboratory (Sport Science Laboratory – Jacques Rogge, Ghent University) on 2 different occasions prior to and following a 6-week training intervention. Subjects completed a maximal ramp incremental (RI) exercise test to assess their responses and adaptations in general cardiorespiratory fitness [gas exchange threshold (GET); peak oxygen uptake, $\dot{\text{V}}\,{\text{O}}_{2}$_peak_] and in tissue oxygenation at the level of the M. Vastus Lateralis and prefrontal cortex. The study intervention itself consisted of 6-week cycling training with several bouts at a work rate corresponding to the Critical Power, as determined prior to the training period from 4 constant work rate trials (CWR trials) to exhaustion.

#### Experimental Testing

The incremental ramp exercise started from 3 min of baseline cycling at 50 W after which work rate (WR) increased continuously (25 W min^–1^). Participants were instructed to keep their cadence between 70 and 80 rpm and strong verbal encouragement was provided throughout the test to ensure maximum effort. The protocol was terminated at voluntary exhaustion or when the subjects’ cadence fell below 70 rpm for more than 5 consecutive seconds. Pulmonary gas exchange ($\dot{\text{V}}\,{\text{O}}_{2}$,$\dot{\text{V}}$CO_2_) was measured breath-by-breath (Jaeger Oxycon Pro, Viasys Healthcare GmbH, Höchberg, Germany) and tissue oxygenation was registered using near-infrared spectroscopy (NIRO-200NX, Hamamatsu Phototonics, Hamamatsu, Japan) at a sampling rate of 0.5 Hz. This device records changes in oxygenated (O_2_Hb), deoxygenated (HHb), and total hemoglobin (totHb) from baseline values (i.e., seated rest) in μmol l^–1^ using the modified Beer Lambert law and TOI employing spatially resolved spectroscopy. Muscle oxygenation (mO_2_Hb, mHHb, mtotHb, and mTOI) was measured at the M. Vastus Lateralis of the right thigh. After shaving and cleaning, the probe was placed longitudinally on the distal section of the muscle belly. Simultaneously, cerebral oxygenation (cO_2_Hb, cHHb, ctotHb, and cTOI) was measured at the level of the right prefrontal cortex between Fp2 en F4 according to the modified international EEG 10-20 system (Robertson and Marino, 2015). Heart rate (HR) was monitored on a beat-by-beat basis (H7 Sensor, Polar, Kempele, Finland). During testing, environmental conditions were kept constant at a room air temperature of 19° and humidity of 50%.

#### Training Period

Six weeks of supervised cycling interval training on the ergometer was completed by all eleven subjects. Training sessions took place three times a week with a total of 18 visits. Each training lasted 49 min and included a 5 min warm-up and a 5 min cooling-down at a WR corresponding to the subject’s GET, as determined from the ramp incremental test. To determine the WR that would elicit a steady state $\dot{\text{V}}\,{\text{O}}_{2}$ corresponding to the GET, the linear $\dot{\text{V}}\,{\text{O}}_{2}$/WR-relationship was shifted to the left to account for the mean response time (MRT; Fontana et al., 2015). The main part of each training was composed of six exercise bouts during which participants cycled at their Critical Power for 4 min, alternated with 3 min of active recovery at the level of the GET. Critical power of the subjects was determined prior to the training intervention from the relationship between work rate and time to exhaustion from four CWR trials to exhaustion at 75, 85, 95, and 105% P_peak_. During training sessions, subjects cycled at a self-selected cadence and HR sensors were worn.

### Data Analysis

Peak power output (P_peak_) and peak heart rate (HR_peak_) were defined as the highest values obtained during the RI test. Breath-by-breath $\dot{\text{V}}\,{\text{O}}_{2}$ data were transformed into 10s values for further analysis. $\dot{\text{V}}\,{\text{O}}_{2}$_peak_ was defined as the highest 30 s average achieved during the test. GET and RCP were determined by four independent researchers. GET was defined as (a) the point where $\dot{\text{V}}$CO_2_ increased disproportionate to $\dot{\text{V}}$O_2_, (b) the first departure from the linear increase in minute ventilation ($\dot{\text{V}}$E), and (c) an increase in $\dot{\text{V}}$E/$\dot{\text{V}}$O_2_ without a simultaneous increase in $\dot{\text{V}}$E/$\dot{\text{V}}$CO_2_ (Beaver et al., 1986). In case the physiologists encountered conflicting results, data were re-evaluated until mutual agreement was reached. RCP was defined as (a) the second departure from the linear increase in $\dot{\text{V}}$E, (b) an increase in $\dot{\text{V}}$E/$\dot{\text{V}}$CO_2_, and (c) a systemic fall in end-tidal PCO_2_ (Whipp et al., 1989).

The NIRS data (O_2_Hb, HHb, tHb, and TOI) were averaged into 10s-bins, expressed as function of power output and analyzed using a double-linear model (Osawa et al., 2011; Bellotti et al., 2013; Boone et al., 2015) (Sigmaplot 13, Systat Software Inc., San Jose, CA, United States). The data set used in this analysis was chosen on visual inspection by three independent researchers and included all data points between the middle portion of the RI test (i.e., the point where the signal showed a systematic linear pattern) and the end of the RI test. Piecewise linear regression analysis was applied and yielded two linear functions (expect for mO_2_Hb in which the signal did not show a clear breakpoint, see “Results” section):

y⁢1=m⁢1×x+b⁢1⁢for⁢x<BP

y⁢2=m⁢2×x+b⁢2⁢for⁢x>BP

where *m* represents the slope and *b* corresponds to the *y*-intercept. Subsequently, a breakpoint (BP) reflecting the intersection of these two linear functions could be determined. The WR at the time-point corresponding to the BPs was adjusted for the $\dot{\text{V}}$O_2_ MRT in each individual in order to account for the kinetics of $\dot{\text{V}}$O_2_ and the delay between muscles and lungs (Boone and Bourgois, 2012; Fontana et al., 2015). The MRT was defined as the time interval between the onset of the RI test and the intersection of the forward extrapolation of the baseline $\dot{\text{V}}$O_2_ and the backward extrapolation of the linear $\dot{\text{V}}$O_2_-time relationship below GET (Fontana et al., 2015). For each subject, the regression line (*y* = *ax* + *b*) of the $\dot{\text{V}}$O_2_/time relationship below GET was calculated. To make sure that the linear increase in $\dot{\text{V}}$O_2_ had already started, the first 2 min of the RI test were omitted from the analysis. Baseline $\dot{\text{V}}$O_2_ was defined as the mean $\dot{\text{V}}$O_2_ during the warm-up phase of the RI test, leaving out the first 90 s and the last 30 s. The individual MRT was then used to align the $\dot{\text{V}}$O_2_ data with the WR data in order to determine the $\dot{\text{V}}$O_2_ at which the BPs occurred. Additionally, the amplitude of mO_2_Hb, mHHb, mtotHb, mTOI, cO_2_Hb, cHHb, ctotHb, and cTOI was calculated as the largest change in the signal from the baseline values. To quantify the change in amplitude (Δ) following the training program the amplitude of the posttest was expressed relative (in %) to the amplitude of the pretest.

### Statistical Analysis

All statistics were performed using SPSS Statistics 23 (IBM Corp., New York, NY, United States). Descriptive data are presented as mean values ± SD for *n* = 11 subjects. The Shapiro-Wilk test indicated that all variables were normally distributed. Paired samples *t*-tests were performed to detect training effects for P_peak_, $\dot{\text{V}}$O_2__peak_, HR_peak_, RER_peak_. Also, the slopes, *y*-intercepts and amplitude of the NIRS-responses were compared using paired samples *t*-tests to identify pre–post-training differences. The breakpoints (BP) in muscle and cerebral oxygenation were compared by means of Repeated Measures ANOVA. Additionally, to assess the relationship between the change in aerobic fitness (i.e., relative increase from pre to post in $\dot{\text{V}}$O_2__peak_) and changes in muscle and cerebral oxygenation (relative change from pre to post in amplitude and slope), a multiple linear regression analysis was performed with the inclusion of the NIRS variables that were affected by the training program. A stepwise backward elimination method was applied in which variables are eliminated that are redundant and/or do not contribute in predicting the outcome variable (i.e., $\dot{\text{V}}$O_2__peak_) and thus, when their contribution to the coefficient of determination (*R*^2^) was not significant. The accuracy of the prediction across independent variables (i.e., contributing NIRS variables) was expressed as adjusted *R*^2^. Additionally, Pearson correlations were calculated between the significant variables of the multiple regression analysis and the relative increase in $\dot{\text{V}}$O_2__peak_ (in %). Statistical significance level was set at *P* < 0.05.

## Results

### General Cardiorespiratory Response

In Table 1 an overview is provided of the general cardiorespiratory response to the ramp incremental tests. The training intervention induced an increase in P_peak_ of 7.9 ± 2.1% (*P* < 0.001), in $\dot{\text{V}}$O_2__peak_ of 7.8 ± 5.0% (*P* < 0.001). RER_peak_ and HR_peak_ did not differ significantly post-training compared to pre-training (*P* = 0.766 and *P* = 0.875, respectively).

**TABLE 1:** Overview of the cardiorespiratory responses to incremental ramp exercise pre- and post-training intervention.

	**Pre**	**Post**	**Δ**
P_peak_ (Watt)	384 ± 36	415 ± 39	31 ± 8^∗^
$\dot{\text{V}}$O_2__peak_ (ml min^–1^)	3967 ± 308	4272 ± 357	305 ± 217^∗^
$\dot{\text{V}}$O_2__peak_ (ml min^–1^ kg^–1^)	52.4 ± 3.5	56.4 ± 3.8	4.0 ± 2.5^∗^
$\dot{\text{V}}$CO_2__peak_ (ml min^–1^)	4959 ± 429	5260 ± 471	301 ± 197^∗^
$\dot{\text{V}}$CO_2__peak_ (ml min^–1^ kg^–1^)	65.5 ± 5.3	69.4 ± 6.2	3.9 ± 2.1^∗^
RER_peak_	1.25 ± 0.06	1.23 ± 0.06	0.01 ± 0.02
HR_peak_ (bts min^–1^)	190 ± 10	190 ± 9	0 ± 3

*Values are mean ± SD. P_*peak*_, peak power output; $\dot{V}$O_2__*peak*_, peak oxygen uptake; $\dot{V}$CO_2peak_, peak carbon dioxide production; RER_*peak*_, peak Respiratory Exchange Ratio; HR_*peak*_, peak heart rate. ^∗^Indicates a significant (*P* < 0.05) increase post-training compared to pre-training.*

### Muscle Oxygenation

**FIGURE 1:**
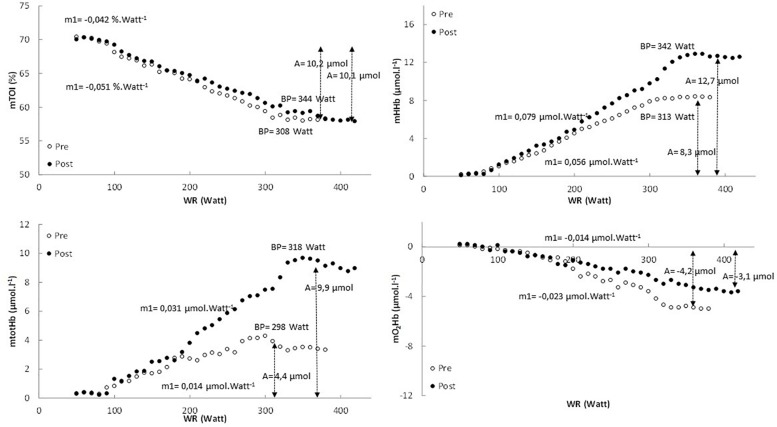
The pattern of mTOI, mHHb, mtotHb, and mO_2_Hb expressed as a function of power output for the pretest (white dots) and posttest (black dots) in a representative subject.

In Figure 1 the pattern of mTOI, mHHb, mtotHb, and mO_2_Hb is presented as function of power output for a representative subject.

In Table 2 an overview is provided of the (double) linear fitting to mHHb, mtotHb, mO_2_Hb, and mTOI. For mTOI, m1 (−16.6 ± 6.4%, *P* = 0.017) was significantly lower and BP was significantly higher (*P* < 0.01) post-training compared to pre-training, whereas A of the response did not differ (*P* = 0.572). The mTOI at baseline cycling (70.1 ± 4.4% vs. 70.2 ± 4.1%, *P* = 0.881), at the BP (58.2 ± 3.4% vs. 58.4 ± 3.2%, *P* = 0.652) and at the end of the exercise test (57.7 ± 3.8% vs. 57.4 ± 3.6%, *P* = 0.857) did not differ between pre- and post-training. For mHHb, both m1 (61.5 ± 28.6%, *P* < 0.01) and A (+40.4 ± 15.8%, *P* < 0.01) were significantly higher post-training compared to pre-training. Also the BP occurred at a higher power output and $\dot{\text{V}}$O_2_ post-training (*P* < 0.001). For mtotHb, m1 (+118.8 ± 57.6%, *P* < 0.001) and A (+125.5 ± 43.1%, *P* < 0.001) were significantly higher post-training and also the BP occurred at a significantly (*P* = 0.02) higher power output and $\dot{\text{V}}$O_2_.

**TABLE 2:** The mean parameters of the (double) linear regression analysis of mHHb, mtotHb, mO_2_Hb, and mTOI to the incremental ramp exercise prior to (pre) and following (post) the training intervention.

	**mHHb**		**mtotHb**		**mO_2_Hb**			**mTOI**	
					
	**Pre**	**Post**	**Pre**	**Post**	**Pre**	**Post**		**Pre**	**Post**
m1 (μmol l^–1^ Watt^–1^)	0.052 ± 0.029	0.084 ± 0.045^∗^	0.016 ± 0.012	0.035 ± 0.019^∗^	−0.022 ± 0.007	−0.014 ± 0.006^∗^	m1 (% Watt^–1^)	0.048 ± 0.012	0.040 ± 0.009^∗^
*y*-intercept1 (μmol l^–1^)	−5.1 ± 4.2	−15.1 ± 8.0^∗^	−0.6 ± 4.6	−3.8 ± 6.1	6.3 ± 3.8	3.8 ± 2.9	*y*-intercept1 (%)	73.2 ± 3.4	72.8 ± 2.8
m2 (μmol l^–1^ Watt^–1^)	−0.001 ± 0.004	−0.002 ± 0.003	−0.03 ± 0.02	−0.02 ± 0.03			m2 (% Watt^–1^)	0.009 ± 0.006	0.015 ± 0.006
*y*-intercept2 (μmol l^–1^)	8.8 ± 3.0	13.0 ± 3.4	11.7 ± 4.8	18.1 ± 6.9			*y*-intercept2 (%)	61.2 ± 4.3	64.1 ± 5.2
BP (Watt)	317 ± 20	342 ± 30^∗^	304 ± 25	330 ± 29^∗^			BP (Watt)	320 ± 23	347 ± 34^∗^
BP (ml min^–1^)	3820 ± 371	4051 ± 390^∗^	3674 ± 392	3969 ± 421^∗^			BP (ml min^–1^)	3847 ± 409	4094 ± 430^∗^
A (μmol l^–1^)	9.4 ± 4.8	13.2 ± 5.3^∗^	4.3 ± 4.1	9.7 ± 5.5^∗^	−6.0 ± 3.2	−4.7 ± 2.9^∗^	A (%)	−12.4 ± 3.1%	−12.8 ± 2.8%

*mHHb, muscle deoxygenated Hb concentration; mtotHb, muscle total Hb concentration; mO_2_Hb, muscle oxygenated Hb concentration; mTOI, muscle tissue oxygenation index; m1, slope of the first part of the double linear model; *y*-intercept 1, *y*-intercept of the first part of the double linear model; m2, slope of the second part of the double linear model; *y*-intercept 2, *y*-intercept of the second part of the double linear model; BP, breakpoint; A, amplitude. ^∗^Indicates a significant (*P* < 0.05) difference between post-training and pre-training.*

The mO_2_Hb response did not show a uniform pattern for all subjects and based on visual inspection not all subjects showed a deflection of mO_2_Hb at high intensities (4 in the pretest and 5 in the posttest). Therefore, mO_2_Hb pattern was analyzed using a linear regression analysis to obtain insight into the slope of the decrease in mO_2_Hb. For mO_2_Hb the slope (m1) (−36.2 ± 17.5%, *P* = 0.017) and the total amplitude (A) (−22.7 ± 10.2%, *P* = 0.024) of the decrease was significantly lower post-training compared to pre-training.

### Cerebral Oxygenation

**FIGURE 2:**
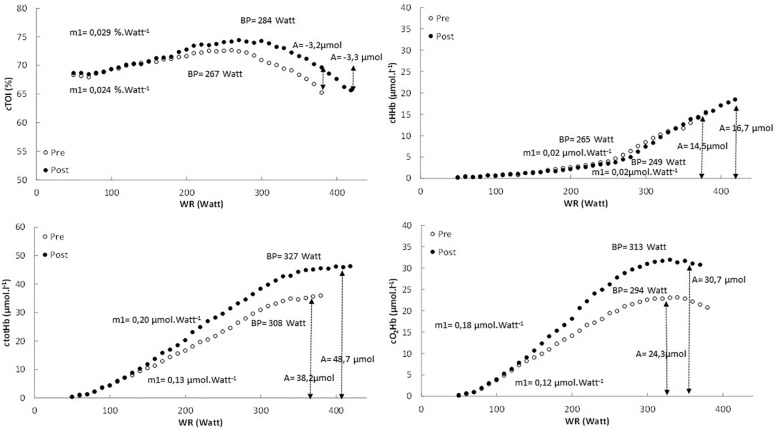
The pattern of cTOI, cHHb, ctotHb, and cO_2_Hb expressed as a function of power output for the pretest (white dots) and posttest (black dots) in a representative subject.

In Figure 2 the pattern of cTOI, cHHb, ctotHb, and cO_2_Hb is presented as function of power output for a representative subject. In Table 3 an overview is provided of the double linear fitting to cHHb, ctotHb, cO_2_Hb, and cTOI. For cTOI, the BP occurred at a higher power output and $\dot{\text{V}}$O_2_ post-training (*P* < 0.001). Although m1 did not differ significantly (*P* = 0.081), cTOI was significantly higher at the power output corresponding to BP (72.6 ± 1.8% vs. 74.6 ± 1.6%, *P* = 0.039). The cTOI at baseline cycling (68.2 ± 3.6% vs. 68.5 ± 4.1%, *P* = 0.592) and the end of the exercise test did not differ (*P* = 0.534) between pre- and post-training (64.3 ± 3.9% vs. 64.8 ± 4.4%, *P* = 0.724). The cTOI at BP (70.8 ± 4.0% vs. 73.5 ± 3.3%, *P* = 0.019) was significantly higher post-training compared to pre-training. For cHHb, only the BP was significantly higher post-training compared to pre-training (*P* < 0.001). For ctotHb, m1 (50.1 ± 14.8%, *P* = 0.023) and A (+26.8 ± 13.6%, *P* = 0.031) were significantly higher post-training and also the BP occurred at a significantly higher power output (*P* = 0.037) and $\dot{\text{V}}$O_2_ (*P* = 0.029). For cO_2_Hb, m1 (+25.0 ± 11.5%, *P* = 0.034) and A (+40.1 ± 18.7%, *P* < 0.001) were significantly higher post-training compared to pre-training and also the BP occurred at a significantly higher (*P* < 0.001) power output and $\dot{\text{V}}$O_2_.

**TABLE 3:** The mean parameters of the double linear regression analysis of cHHb, ctotHb, cO_2_Hb and cTOI to the incremental ramp exercise prior to (pre) and following (post) the training intervention.

	**cHHb**		**ctotHb**		**cO_2_Hb**			**cTOI**	
					
	**Pre**	**Post**	**Pre**	**Post**	**Pre**	**Post**		**Pre**	**Post**
m1 (μmol l^–1^ Watt^–1^)	0.02 ± 0.01	0.02 ± 0.01	0.12 ± 0.06	0.18 ± 0.05^∗^	0.16 ± 0.04	0.20 ± 0.07^∗^	m1 (% Watt^–1^)	0.025 ± 0.008	0.030 ± 0.011
*y*-intercept1 (μmol l^–1^)	−2.1 ± 1.9	−3.4 ± 2.2	−9.8 ± 5.2	−15.1 ± 6.2	−22.7 ± 11.4	−31.4 ± 11.7^∗^	*y*-intercept1 (%)	65.6 ± 4.4	66.2 ± 4.0
m2 (μmol l^–1^ Watt^–1^)	0.10 ± 0.03	0.10 ± 0.03	0.05 ± 0.02	0.05 ± 0.03	−0.05 ± 0.05	−0.07 ± 0.05	m2 (% Watt^–1^)	−0.067 ± 0.022	−0.069 ± 0.027
*y*-intercept2 (μmol l^–1^)	−15.5 ± 5.2	−17.9 ± 6.2	17.1 ± 7.8	27.8 ± 9.1	38.2 ± 10.7	41.4 ± 12.2	*y*-intercept2 (%)	92.6 ± 5.1	97.8 ± 6.1
BP (Watt)	251 ± 25	270 ± 33^∗^	312 ± 28	325 ± 32^∗^	289 ± 27	311 ± 25^∗^	BP (Watt)	258 ± 22	275 ± 27^∗^
BP (ml min^–1^)	3137 ± 385	3368 ± 402^∗^	3781 ± 441	3923 ± 381^∗^	3515 ± 379	3803 ± 337^∗^	BP (ml min^–1^)	3207 ± 380	3415 ± 419^∗^
A (μmol l^–1^)	16.2 ± 4.8	18.5 ± 5.3	36.5 ± 7.1	46.3 ± 6.8	22.0 ± 10.0	30.8 ± 13.1^∗^	A (%)	−4.0 ± 2.9%	−4.0 ± 3.1%

*cHHb, cerebral deoxygenated Hb concentration; ctotHb, cerebral total Hb concentration; cO_2_Hb, cerebral oxygenated Hb concentration; cTOI, cerebral tissue oxygenation index; m1, slope of the first part of the double linear model; *y*-intercept 1, *y*-intercept of the first part of the double linear model; m2, slope of the second part of the double linear model; *y*-intercept 2, *y*-intercept of the second part of the double linear model; BP, breakpoint; A, amplitude. ^∗^Indicates a significant (*P* < 0.05) difference between post-training and pre-training.*

### Breakpoints in Muscle and Cerebral Oxygenation

The statistical analysis revealed that there was no interaction effect (*P* = 0.744) indicating that the evolution of the BPs over the training period did not differ. However, there was a specific order in the occurrence of the BPs (expressed in ml min^–1^). BPs of cHHb and cTOI occurred at a significantly (*P* < 0.05) lower $\dot{\text{V}}$O_2_ compared to the other BPs, whereas BPs of mHHb and mTOI occurred at a significantly (*P* < 0.05) higher $\dot{\text{V}}$O_2_ compared to mtotHb, ctotHb and cO_2_Hb. Additionally, there was a significant main effect of training intervention (*P* < 0.001) indicating that all BPs shifted to a higher $\dot{\text{V}}$O_2_ post-training compared to pre-training.

### $\dot{\text{V}}$O_2__peak_ vs. Muscle and Cerebral Oxygenation

**FIGURE 3:**
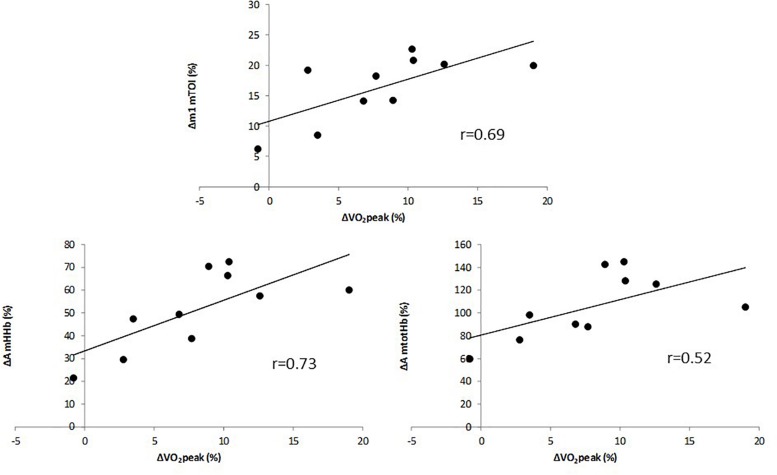
Correlation between relative change in VO_2__peak_ and relative change in the slope (m1) of mTOI **(upper panel)**, and amplitude of mHHb **(lower left panel)** and mtotHb **(lower right panel)**.

The multiple linear regression analysis revealed that only the change in A of mHHb, the change in A of mtotHb and the change in m1 of mTOI contributed significantly to the relative increase in $\dot{\text{V}}$O_2__peak_ (Adjusted *R*^2^ = 0.68). Change in cerebal oxygenation parameters did not significantly add to the multiple regression analysis (*P* > 0.05). The relative increase in $\dot{\text{V}}$O_2__peak_ (in %) was correlated to the relative change (in %) in m1 of mTOI (Figure 3, upper panel) (*r* = 0.69, *P* = 0.011) and, in the amplitude of mHHb (Figure 3, lower left panel) (*r* = 0.73, *P* = 0.003) and mtotHb (Figure 3, lower right panel) (*r* = 0.52, *P* = 0.021). The improvement in $\dot{\text{V}}$O_2__peak_ was not correlated with any relative changes in m1 and A of cerebral oxygenation parameters.

## Discussion

In the present study, the effects of a training intervention on muscle and brain oxygenation during incremental exercise were assessed. It was observed that aerobic interval training affects the patterns of muscle and cerebral oxygenation to incremental ramp exercise, in concert with an improvement with aerobic fitness indices (e.g., $\dot{\text{V}}$O_2__peak_). More specifically, at the level of the muscle, it was found that mTOI decreased at a slower rate following the training intervention. The amplitude of mHHb and mtotHb increased from pre- to post-training, supporting an increased capacity for microvascular O_2_ extraction in combination with an improved O_2_ availability. Additionally, it was found that also cerebral O_2_Hb and totHb displayed a steeper increase at moderate intensities and increased to a higher amplitude, resulting in an improved cerebral oxygenation (cTOI) throughout the test. Finally, it was found that the increase in $\dot{\text{V}}$O_2__peak_ was predominantly related to changes in muscle oxygenation (i.e., decrease in the slope of mTOI, increase in amplitude of mHHb and mtotHb) and not to changes in cerebral oxygenation.

### Effect of Training on Muscle Oxygenation

The effects of a training intervention on muscle oxygenation responses, assessed with NIRS, to incremental exercise are scarcely documented. In the present study an aerobic interval training program, consisting of work bouts at the level of the critical power (i.e., the boundary between the heavy and the severe intensity domain), resulted in an improved muscle oxygenation throughout the incremental exercise test, as can be deducted from the slower decrease in mTOI post-training. As mtotHb reflects the amount of Hb under the NIRS probe, the increase in the slope and amplitude of mtotHb indicates that the O_2_ availability at the muscle level throughout the incremental exercise has improved. Since the change with the interval training in mHHb, which can be considered to reflect microvascular O_2_ extraction, is less pronounced than the change in mtotHb, this will result in a slower decrease in overall tissue oxygenation (i.e., mTOI). The increase in O_2_ availability (i.e., higher slope and amplitude of mtotHb) has likely resulted from training-induced adaptations in O_2_ diffusive capacity and/or convective O_2_ supply. The O_2_ diffusing capacity is dictated predominantly by capillary hematocrit and the volume density of red blood cell-flowing capillaries (Davis and Barstow, 2013). The strong increase in mtotHb following interval training is thus, likely in part the result of an improvement in capillary density (and thus capillary-to-muscle fiber ratio) and adaptations in vascular control inducing a local redistribution of blood flow to strongly recruited areas (Laughlin and Roseguini, 2008). Next to these adjustments at the level of the microcirculation, affecting the O_2_ diffusing capacity, also functional and structural adaptations in the conduit arteries (Dinenno et al., 2001; Rowley et al., 2011; Green et al., 2017) and with regards to cardiac output (MacInnis and Gibala, 2017), can improve the convective O_2_ supply. It has been shown, that there is a certain time course in the adaptations in the different vascular properties, with rapid initial improvements in vascular dilation function to normalize shear stress during exercise bouts. Afterward, a more prolonged training program will induce more permanent structural enlargements (Green et al., 2017). Also, at the level of the microcirculation the adaptations have been reported to occur at an early stage during a training program (Duscha et al., 2011). In this context, it would have been interesting to observe the changes in NIRS responses also in the middle of the training program (i.e., after 3 weeks), next to those at the end (i.e., after 6 weeks) to obtain information on the time course of the adaptations in O_2_ diffusive capacity and convective O_2_ supply.

Additionally, the amplitude of mHHb, reflecting microvascular O_2_ extraction, strongly increased following the training intervention. This response indicates that structural and/or functional adaptations have been induced at the level of the skeletal muscles. In this context Jacobs et al. (2013) have shown that the increase in exercise performance with high-intensity interval training is strongly linked to an increase in skeletal muscle mitochondrial content and function, which has been documented as a general effect of high-intensity interval training (Dinenno et al., 2001). This will in turn affect the driving O_2_ pressure gradient between capillaries and muscle with a consequent increase in O_2_ extraction capacity. Previous studies in healthy (Murias et al., 2010; Jacobs et al., 2013; Prieur and Mucci, 2013) and patient populations (Fu et al., 2016; Takagi et al., 2016) have found a similar effect on mHHb following training, whereas cross-sectional studies have also shown a positive relationship between $\dot{\text{V}}$O_2__peak_ and the amplitude of the mHHb response during exercise (Okushima et al., 2016).

Interestingly, the change in $\dot{\text{V}}$O_2__peak_ showed to be related to the changes in muscle oxygenation parameters. Individuals that could maintain mTOI at a higher level compared to pre-training and that could enhance mHHb (microvascular O_2_ extraction) to a higher extent showed the strongest improvements in $\dot{\text{V}}$O_2__peak_. The observation that mTOI at the breakpoint, where a leveling-off in mTOI occurs, and at the end of the incremental exercise were similar pre-training compared to post-training suggests that a critical level of mTOI dictates the termination of the incremental exercise test or at least triggers a cascade that leads to this termination.

### Effect of Training on Cerebral Oxygenation

Also at cerebral level the training-induced adaptations support an enhanced oxygenation level. The slope (m1) of cTOI is slightly but not significantly steeper, cTOI at the breakpoint is higher, and also the observation that the slope and amplitude of cO_2_Hb and ctotHb are higher support an improved O_2_ availability throughout the incremental exercise. It has been found (e.g., Peltonen et al., 2009; Rooks et al., 2010; Oussaidene et al., 2013), also in the present study, that cO_2_Hb and ctotHb increase up to a high intensity (∼75% P_peak_), where the response first levels off and then starts to decrease. cTOI follows a similar pattern albeit that the breakpoint occurs at a slightly lower intensity. These responses indicate that at high intensities there is a mismatch between O_2_ supply and O_2_ demand at the level of the prefrontal cortex. The occurrence of the breakpoint in cO_2_Hb and ctotHb, following the increase at low to moderate intensities, can be related to the pattern of cerebral blood flow during incremental exercise which depends on the exercise intensity (Jorgensen et al., 1992; Gonzalez-Alonso et al., 2004; Querido and Sheel, 2007). Cerebral blood flow is tightly regulated by the cerebrovascular responsiveness to alterations in partial pressures of O_2_ (PaO_2_) and CO_2_ (PaCO_2_) (Paulsen et al., 1990). At moderate intensities cerebral blood flow increases in relation to the increase in cardiac output. Once the GET is exceeded, PaCO_2_ increases and induces a vasodilation resulting in a more pronounced blood flow to the brain. It appears from the higher slope and amplitude of cO_2_Hb and ctotHb that cerebral blood flow is enhanced following interval training, dictated by a commonly reported increase in cardiac output following interval training (MacInnis and Gibala, 2017) and/or redistribution of the blood flow favoring the prefrontal cortex. These results correspond to the study of Oussaidene et al. (2015) were similar responses were found in trained vs. untrained subjects and indicate that the O_2_ supply exceeded the demand more following the training intervention. However, at the respiratory compensation point the PaCO_2_ is reduced (i.e., hypocapnia) due to the ventilatory response to the metabolic acidosis and this will reduce cerebral blood flow. This mechanistic link between RCP and the BP in cO_2_Hb also explain the tight relationship between the two parameters observed in the present study.

Given this decrease in cerebral oxygenation at a given intensity during incremental exercise, the role of the brain (i.e., cerebral oxygenation) in the termination of incremental exercise has been put forward (e.g., Subudhi et al., 2008). The decrease in cO_2_Hb, ctotHb, and cTOI following the breakpoint (i.e., at high intensities) in cerebral oxygenation might have a negative impact on central motor command (Ide and Secher, 2000; Subudhi et al., 2007; Rupp and Perrey, 2008) and as such set a limitation to the maximal exercise performance. The present study showed that the changes in cerebral oxygenation parameters (i.e., higher cTOI at BP, higher slope and amplitude of cO_2_Hb and ctotHb) following the training intervention were not related to the changes in $\dot{\text{V}}$O_2__peak_. This indicates that cerebral oxygenation *per se* does not dictate the termination of the incremental exercise test, in contrast to the suggestion of Robertson and Marino (2016).

The results of the present study, however, suggest that a tight coupling exists between the muscle and brain during incremental exercise. The breakpoints in the muscle and cerebral oxygenation parameters occur in a specific order which is unchanged by the interval training program. First cHHb and cTOI start to level off probably as a consequence of the higher cerebral metabolic load to increase motor command to fast twitch fibers in response to the increase in power output and to account for fatigue in earlier recruited muscle fibers. The progressive recruitment of these less oxidative fibers enhances the occurrence of a metabolic acidosis, which has an impact on the ventilatory responses (RCP). As indicated above, this will reduce PaCO_2_ which will in turn affect cerebral blood flow (Paulsen et al., 1990) and thus, cO_2_Hb and ctotHb. The origin of the plateau in mHHb (and here also mTOI) and possible link with the cerebral oxygenation responses is currently unclear. Although originally the plateau in mHHb was considered as a leveling-off in O_2_ extraction, recent studies have shown an O_2_ extraction reserve following incremental ramp exercise when a blood flow occlusion is applied (Inglis et al., 2017; Iannetta et al., 2018). It is argued that the leveling off in mHHb near the end of exercise is related to locally released vasoactive compounds (H^+^, ATP, lactate, K^+^) triggering a local vasodilation and thus a redistribution of the blood flow. In order to establish a mechanistic link between muscle and cerebral oxygenation it would be interesting to directly measure the effect of training on blood gas (arterial O_2_ and CO_2_ pressures) and metabolite (e.g., lactate, H^+^) parameters.

## Conclusion

The present study shows that aerobic interval training impacts both muscle and brain oxygenation, in concert with an increase in aerobic fitness. More specifically, the training program slowed the decrease in mTOI and increased the amplitude of mtotHb and mHHb, pointing at an improved O_2_ availability and O_2_ extraction capacity, respectively. Also the amplitude of cerebral O_2_Hb and totHb was increased, suggesting an enhanced brain perfusion at high intensities. The observation that the increase in $\dot{\text{V}}$O_2__peak_ correlated with the changes in the slope of mTOI and, amplitudes of both muscle HHb and totHb, but not to those of cerebral oxygenation, indicate that brain oxygenation *per se* will not be a primary limiting factor to incremental exercise tests.

## Data Availability

The datasets generated for this study are available on request to the corresponding author.

## Author Contributions

KC, KV, JGB, and JB: study design. KC, KV, and JB: data collection. KC, KV, AM, VN, SP, and JB: data analysis. KC, KV, AM, VN, SP, JGB, and JB: data interpretation. KC, VN, SP, and JB: writing manuscript. AM, VN, SP, and JGB: revising manuscript.

## Conflict of Interest Statement

The authors declare that the research was conducted in the absence of any commercial or financial relationships that could be construed as a potential conflict of interest.

## References

[B1] Beaver W. L. Wasserman K. Whipp B. J. (1986). A new method for detecting anaerobic threshold by gas exchange. *J. Appl. Physiol.* 60 2020–2027. 10.1152/jappl.1986.60.6.2020 3087938

[B2] Bellotti C. Calabria E. Capelli C. Pogliaghi S. (2013). Determination of maximal lactate steady state in healthy adults: can NIRS help? *Med. Sci. Sports Exerc.* 45 1208–1216. 10.1249/MSS.0b013e3182828ab2 23274611

[B3] Bhambhani Y. Malik R. Mookerjee S. (2007). Cerebral oxygenation declines at exercise intensities above the respiratory compensation point. *Resp. Physiol. Neurobiol.* 156 196–202. 10.1016/j.resp.2006.08.009 17045853

[B4] Boone J. Barstow T. J. Celie B. Prieur F. Bourgois J. (2015). The impact of pedal rate on muscle oxygenation, muscle activation and whole-body ? during ramp exercise in healthy subjects. *Eur. J. Appl. Physiol.* 115 57–70. 10.1007/s00421-014-2991-x 25204279

[B5] Boone J. Bourgois J. (2012). The oxygen uptake response to incremental ramp exercise. *Sports Med.* 42 511–526. 10.2165/11599690-000000000-00000 22571502

[B6] Boone J. Koppo K. Barstow T. J. Bouckaert J. (2009). Pattern of deoxy[Hb+Mb] during ramp cycle exercise: influence of aerobic fitness status. *Eur. J. Appl. Physiol.* 105 851–859. 10.1007/s00421-008-0969-2 19130074

[B7] Boone J. Vandekerckhove K. Coomans I. Prieur F. Bourgois J. (2016). An integrated view on the oxygenation responses to incremental exercise at the brain, the locomotor and respiratory muscles. *Eur. J. Appl. Physiol.* 116 2085–2102. 10.1007/s00421-016-3468-x 27613650

[B8] Davis M. L. Barstow T. J. (2013). Estimated contribution of hemoglobin and myoglobin to near infrared spectroscopy. *Respir. Physiol. Neurobiol.* 186 180–187. 10.1016/j.resp.2013.01.012 23357615

[B9] Delorey D. S. Kowalchuk J. M. Paterson D. M. (2003). Relationship between pulmonary O2 uptake kinetics and muscle deoxygenation during moderate-intensity exercise. *J. Appl. Physiol.* 95 113–120. 10.1152/japplphysiol.00956.2002 12679363

[B10] Dinenno F. A. Tanaka H. Monahan K. D. Clevenger C. M. Eskurza I. DeSouza C. A. (2001). Regular endurance exercise induces expansive arterial remodeling in the trained limbs of healthy men. *J. Physiol.* 534 287–295. 10.1111/j.1469-7793.2001.00287.x 11433009 PMC2278690

[B11] Duscha B. D. Robbins J. L. Jones W. S. Kraus W. E. Lye R. J. Sanders J. M. (2011). Angiogenesis in skeletal muscle precede improvements in peak oxygen uptake in peripheral artery disease patients. *Arterioscler. Thromb. Vasc. Biol.* 31 2742–2748. 10.1161/ATVBAHA.111.230441 21868709 PMC3578302

[B12] Ferreira L. F. Koga S. Barstow T. J. (2007). Dynamics of non-invasively estimated microvascular O2 extraction during ramp exercise. *J. Appl. Physiol.* 103 1999–2004. 10.1152/japplphysiol.01414.2006 17823295

[B13] Fontana F. Y. Keir D. A. Bellotti C. De Roia G. F. Murias J. M. Pogliaghi S. (2015). Determination of respiratory compensation point in healthy adults: can non-invasive near-infrared spectroscopy help? *J. Sci. Med. Sport* 18 590–595. 10.1016/j.jsams.2014.07.016 25153251

[B14] Fu T. Yang N. Wang C. Cherng W. Chou S. Pan T. (2016). Aerobic interval training elicits different hemodynamic adaptations between heart failure patients with preserved and reduced ejection fraction. *Am. J. Phys. Med. Rehabil.* 95 15–27. 10.1097/PHM.0000000000000312 26053189

[B15] Gifford J. R. Garten R. S. Nelson A. D. Trinity J. D. Layec G. Witman M. A. H. (2016). Symmorphosis and skeletal muscle O2max: in vivo and in vitro measures reveal differing constraints in the exercise-trained and untrained human. *J. Physiol.* 594 1741–1751. 10.1113/JP271229 26614395 PMC4799962

[B16] Gonzalez-Alonso J. Dalsgaard M. K. Osada T. Volianitis S. Dawson E. A. Yoshiga C. C. (2004). Brain and central haemodynamics and oxygenation during maximal exercise in humans. *J. Physiol.* 557 331–342. 10.1113/jphysiol.2004.060574 15004212 PMC1665053

[B17] Grassi B. Pogliaghi S. Rampichini S. Quaresima V. Ferrari M. Marconi C. (2003). Muscle oxygenation and pulmonary gas exchange kinetics during cycle exercise on-transitions in humans. *J. Appl. Physiol.* 95 149–158. 10.1152/japplphysiol.00695.2002 12611769

[B18] Green D. J. Hopman M. T. E. Padilla J. Laughlin M. H. Thijssen D. H. J. (2017). Vascular adaptation to exercise in humans: role of hemodynamic stimuli. *Physiol. Rev.* 97 495–528. 10.1152/physrev.00014.2016 28151424 PMC5539408

[B19] Iannetta D. Okushima D. Inglis E. C. Kondo N. Murias J. M. Koga S. (2018). Blood flow occlusion-related O2 extraction “reserve” is present in different muscles of the quadriceps but greater in deeper regions after ramp-incremental test. *J. Appl. Physiol.* 125 313–319. 10.1152/japplphysiol.00154.2018 29722622

[B20] Ide K. Secher N. H. (2000). Cerebral blood flow and metabolism during exercise. *Progr. Neurobiol.* 61 397–414. 10.1016/s0301-0082(99)00057-x 10727781

[B21] Inglis E. C. Iannetta D. Murias J. M. (2017). The plateau in the NIRS-derived [HHb] signal near the end of a ramp incremental exercise test does not indicate the upper limit of O2 extraction in the vastus lateralis. *Am. J. Phys. Reg. Integr. Comp. Phys.* 313 R723–R729. 10.1152/ajpregu.00261.2017 28931547 PMC5814694

[B22] Jacobs R. A. Flück D. Bonne R. C. Bürgi S. Christensen P. M. Toigo M. (2013). Improvements in exercise performance with high-intensity interval training coincide with an increase in skeletal muscle mitochondrial content and function. *J. Appl. Physiol.* 115 785–793. 10.1152/japplphysiol.00445.2013 23788574

[B23] Jorgensen L. G. Perko G. Secher N. H. (1992). Regional cerebral artery mean flow velocity and blood flow during dynamic exercise in humans. *J. Appl. Physiol.* 73 1825–1830. 10.1152/jappl.1992.73.5.1825 1474058

[B24] Laughlin M. H. Roseguini B. (2008). Mechanisms for exercise training-induced increases in skeletal muscle blood flow capacity: differences with interval sprint training versus aerobic endurance training. *J. Physiol. Pharmacol.* 59 71–88. 19258658 PMC2654584

[B25] MacInnis M. J. Gibala M. J. (2017). Physiological adaptations to interval training and the role of exercise intensity. *J. Physiol.* 595 2915–2930. 10.1113/JP273196 27748956 PMC5407969

[B26] Mancini D. M. Bolinger L. Li H. Kendrick K. Chance B. Wilson J. R. (1994). Validation of near-infrared spectroscopy in humans. *J. Appl. Physiol.* 77 2740–2747. 10.1152/jappl.1994.77.6.2740 7896615

[B27] Murias J. M. Kowalchuk J. M. Paterson D. H. (2010). Time course and mechanisms of adaptations in cardiorespiratory fitness with endurance training in older and young men. *J. Appl. Physiol.* 108 621–627. 10.1152/japplphysiol.01152.2009 20056848

[B28] Nielsen H. B. Boushel R. Madsen P. Secher N. H. (1999). Cerebral desaturation during exercise reversed by O2 supplementation. *Am. J. Physiol.* 277 1045–1052. 10.1152/ajpheart.1999.277.3.H1045 10484427

[B29] Okushima D. Poole D. C. Barstow T. J. Rossiter H. B. Kondo N. Bowen S. T. (2016). Greater O2peak is correlated with greater skeletal muscle deoxygenation amplitude and hemoglobin concentration within individual muscles during ramp-incremental cycle exercise. *Phys. Rep.* 4:e13065. 10.14814/phy2.13065 27986837 PMC5260088

[B30] Osawa T. Kime R. Hamaoka T. Katsamura T. Yamamoto M. (2011). Attenuation of muscle deoxygenation precedes EMG threshold in normoxia and hypoxia. *Med. Sci. Sports Exerc.* 43 1406–1413. 10.1249/MSS.0b013e3182100261 21266933

[B31] Oussaidene K. Prieur F. Bougault V. Borel B. Matran R. Mucci P. (2013). Cerebral oxygenation during hyperoxia-induced increase in exercise tolerance for untrained men. *Eur. J. Appl. Physiol.* 113 2047–2056. 10.1007/s00421-013-2637-4 23579360

[B32] Oussaidene K. Prieur F. Tagougui S. Abaidia A. Matran R. Mucci P. (2015). Aerobic fitness influences cerebral oxygenation response to maximal exercise in healthy subjects. *Resp. Physiol. Neurobiol.* 205 53–60. 10.1016/j.resp.2014.10.009 25461626

[B33] Paulsen O. B. Strandgaard S. Edvinsson L. (1990). Cerebral autoregulation. *Cerebrovasc. Brain Metab. Rev.* 2 161–192. 2201348

[B34] Peltonen J. E. Paterson D. H. Shoemaker J. K. Delorey D. S. Dumanoir G. R. Petrella R. J. (2009). Cerebral and muscle deoxygenation, hypoxic ventilatory chemosensitivity and cerebrovascular responsiveness during incremental exercise. *Resp. Physiol. Neurobiol.* 169 24–35. 10.1016/j.resp.2009.08.013 19729079

[B35] Prieur F. Mucci P. (2013). Effect of high-intensity interval training on the profile of muscle deoxygenation heterogeneity during incremental exercise. *Eur. J. Appl. Physiol.* 113 249–257. 10.1007/s00421-012-2430-9 22677918

[B36] Querido J. S. Sheel A. W. (2007). Regulation of cerebral blood flow during exercise. *Sports Med.* 37 765–782. 10.2165/00007256-200737090-00002 17722948

[B37] Racinais S. Buchheit M. Girard O. (2014). Breakpoints in ventilation, cerebral and muscle oxygenation, and muscle activity during incremental cycling exercise. *Front. Physiol.* 5:142. 10.3389/fphys.2014.00142 24782786 PMC3990045

[B38] Robertson C. V. Marino F. E. (2015). Prefrontal and motor cortex EEG responses and their relationship to ventilatory responses during exhaustive incremental exercise. *Eur. J. Appl. Physiol.* 115 1939–1948. 10.1007/s00421-015-3177-x 25917836

[B39] Robertson C. V. Marino F. E. (2016). A role for the prefrontal cortex in exercise tolerance and termination. *J. Appl. Physiol.* 120 464–466. 10.1152/japplphysiol.00363.2015 26404617

[B40] Rooks C. R. Thom N. J. McCully K. K. Dishman R. K. (2010). Effects of incremental exercise on cerebral oxygenation measured by near-infrared spectroscopy: a systematic review. *Prog. Neurobiol.* 92 134–150. 10.1016/j.pneurobio.2010.06.002 20542078

[B41] Rowley N. J. Dawson E. A. Birk G. K. Cable N. T. George K. Whyte G. (2011). Exercise and arterial adaptation in humans: uncoupling localized and systemic effects. *J. Appl. Physiol.* 110 1190–1195. 10.1152/japplphysiol.01371.2010 21350023

[B42] Rupp T. Perrey S. (2008). Prefrontal cortex oxygenation and neuromuscular responses to exhaustive exercise. *Eur. J. Appl. Physiol.* 102 153–163. 10.1007/s00421-007-0568-7 17882449

[B43] Spencer M. D. Murias J. M. Paterson D. H. (2012). Characterizing the profile of muscle deoxygenation during ramp incremental exercise in young men. *Eur. J. Appl. Physiol.* 112 3349–3360. 10.1007/s00421-012-2323-y 22270488

[B44] Subudhi A. W. Dimmen A. C. Roach R. C. (2007). Effects of acute hypoxia on cerebral and muscle oxygenation during incremental exercise. *J. Appl. Physiol.* 103 177–183. 10.1152/japplphysiol.01460.2006 17431082

[B45] Subudhi A. W. Lorenz M. C. Fulco C. S. Roach R. C. (2008). Cerebrovascular responses to incremental exercise during hypobaric hypoxia: effect of oxygenation on maximal performance. *Am. J. Physiol. Heart Circ. Physiol.* 294 H164–H171. 10.1152/ajpheart.01104.2007 18032522

[B46] Takagi S. Murase N. Kime R. Niwayama M. Osada T. Katsumura T. (2016). Aerobic training enhances muscle deoxygenation in early post-myocardial infarction. *Eur. J. Appl. Physiol.* 116 673–685. 10.1007/s00421-016-3326-x 26759155 PMC4819748

[B47] Whipp B. J. Davis J. A. Wasserman K. (1989). Ventilatory control of the’isocapnic buffering’ region in rapidly-incremental exercise. *Respir. Physiol.* 76 357–367. 10.1016/0034-5687(89)90076-5 2501844

